# Influence of COVID-19 pandemic on the decision making of patients in undergoing gamma knife radiosurgery

**DOI:** 10.1186/s40001-022-00859-w

**Published:** 2022-10-29

**Authors:** Chiung-Chyi Shen, Rong-San Jiang, Men-Yin Yang, Weir-Chiang You, Ming-Hsi Sun, Meei-Ling Sheu, Liang-Yi Pan, Jason Sheehan, Hung-Chuan Pan

**Affiliations:** 1 Department of Neurosurgery, Taichung Veterans General Hospital, Taichung, Taiwan; 2 Department of Medical Research, Taichung Veterans General Hospital, Taichung, Taiwan; 3 Department of Radiation Oncology, Taichung Veterans General Hospital, Taichung, Taiwan; 4 Institute of Biomedical Science, National Chung-Hsin University, Taichung, Taiwan; 5 Faculty of Medicine, Kaohsiung Medical University, Kaohsiung, Taiwan; 6 Department of Neurosurgery, University of Virginia, Charlottesville, VA USA; 7 Department of Medical Research and Neurosurgery, Taichung Veterans General Hospital, 1650 Taiwan Boulevard Sec.4, 40705 Taichung, Taiwan; 8 Rong Hsing Research Center for Translational Medicine, National Chung Hsing University, Taichung, Taiwan

**Keywords:** Gamma knife, Radiosurgery, Decision making, COVID-19, Vaccination

## Abstract

**Purpose:**

Gamma knife radiosurgery (GK) is a commonly used approach for the treatment of intracranial lesions. Its radiation response is typically not immediate, but delayed. In this study, we analyzed cases from a prospectively collected database to assess the influence of COVID-19 pandemic on the decision making in patients treated by gamma knife radiosurgery.

**Methods:**

From January 2019 to August 2021, 540 cases of intracranial lesions were treated by GK with 207 cases before COVID-19 pandemic as a control. During the COVID-19 pandemic, 333 cases were similarly treated on patients with or without the COVID-19 vaccination. All the GK treated parameters as well as time profile in the decision making were analyzed. The parameters included age, sex, characteristic of lesion, targeted volume, peripheral radiation dose, neurological status, Karnofsky Performance Status (KPS), time interval from MRI diagnosis to consultation, time interval from the approval to treatment, frequency of outpatient department (OPD) visit, and frequency of imaging follow-up.

**Results:**

Longer time intervals from diagnosis to GK consultation and treatment were found in the pandemic group (36.8 ± 25.5/54.5 ± 27.6 days) compared with the pre-COVID control (17.1 ± 22.4/45.0 ± 28.0 days) or vaccination group (12.2 ± 7.1/29.6 ± 10.9 days) (*p* < 0.001, and *p* < 0.001, respectively). The fewer OPD visits and MRI examinations also showed the same trends. High proportion of neurological deficits were found in the pandemic group (65.4%) compared with the control (45.4%) or vaccination group (58.1%) (*p* < 0.001). The Charlson comorbidity in the pandemic group was 3.9 ± 3.3, the control group was 4.6 ± 3.2, and the vaccination group was 3.1 ± 3.1. There were similar inter-group difference (*p* < 0.001). In multiple variant analyses, longer time intervals from the diagnosis to consultation or treatment, OPD frequency and MRI examination were likely influenced by the status of the COVID-19 pandemic as they were alleviated by the vaccination.

**Conclusions:**

The decision making in patients requiring gamma knife treatment was most likely influenced by the status of the COVID-19 pandemic, while vaccination appeared to attenuate their hesitant behaviors. Patients with pre-treatment neurological deficits and high co-morbidity undergoing the gamma knife treatment were less affected by the COVID-19 pandemic.

**Supplementary Information:**

The online version contains supplementary material available at 10.1186/s40001-022-00859-w.

## Introduction

Gamma knife radiosurgery is an effective treatment for many intracranial lesions, such as benign or malignant tumor, vascular malformation and functional disorder [1–9]. The decision to pursue treatment and the interval between that affirmative decision and treatment is often affected for the patient concerns including insurance policy, neurological status, patient preference, and severity of the illness.

Coronavirus (COVID-19) originated from the city of Wuhan in the Hubei province of China in late 2019, spreading to Europe, America, and Asia leading a global pandemic [10]. The projected global death is estimated to be 2 million [11]. In response to the pandemic, sweeping changes were enacted including social distancing, school closures, mandatory masking in some states, and telecommuting [12]. As an essential sector of health care, the gamma knife radiosurgery was also affected. Guidelines were quickly put in place by stereotactic radiosurgery (SRS) in terms of patient workflow, delaying care when possible, and use of personal protective equipment (PPE) [13]. Modifications, such as reducing the number of fractions or frequency of frame-based treatment were made to minimize the contacts of the patients and staff but without affecting treatment efficacy [14–16].

Since its emergence, the SARS-CoV-2 virus has continued to evolve, WHO has so far designated 5 variants of SARS-CoV-2 as Variants of Concern (VOC), namely, Alpha, Beta, Gamma, Delta and Omicron, since they have great impacts regarding transmission, disease severity, and the capacity for immune escape. While the Omicron variant is spreading rapidly across the world, the evolution of SARS-CoV-2 is expected to continue. Omicron is unlikely to be the last VOC. The TAG-CO-VAC is developing a framework to analyze the evidence on emerging VOCs that would trigger a recommendation to change COVID-19 vaccine strain composition, advising WHO on updated vaccine compositions. This framework considers the global spread and transmissibility, clinical severity, genetic, antigenic and phenotypic characteristics of the VOC, including capacity for immune escape and assessment of vaccine effectiveness.

Therapeutic benefits of Gamma knife present in a delayed fashion. The longer time windows exist for patients to decide on their treatment strategy especially during the COVID-19 pandemic without or with vaccination. In this study, we analyzed cases from a prospectively collected database to assess the influence of COVID-19 pandemic on the decision making in those patients treated by gamma knife radiosurgery including time interval from diagnosis to consultation, time interval from the diagnosis to GK treatment, frequency of OPD visit (time/year), and frequency of brain MRI imaging follow-up (time/year) and the alteration in the tumor volume and numbers.

## Materials and methods

### Patient population

A total 540 cases of intracranial lesions were treated by GKRS for our analysis from January 2019 to August 2021, there were for analysis 207 cases before the COVID-19 pandemic as the control (Control group), and 333 cases during the COVID-19 pandemic either without (185 cases) (Pandemic group) or with patient vaccination (148 cases) (Vaccination group). Their data for analysis were obtained from the medical chart and data bank of the gamma knife center. Those patients diagnosed with trigeminal neuralgia or functional disorders were excluded from analysis. Patient parameters included the following: age, sex, characteristic of lesion either benign or malignance, targeted volume (TV), peripheral radiation dose, number of lesions, presence of neurological deficits, KPS, ECOG, time interval from MRI diagnosis to consultation, time interval from the approval to treatment, frequency of OPD visit (time/year), and frequency of brain MRI imaging follow-up (time/year). We define the period of pandemic according to the announcement of WHO and CDC of Taiwan to start the quarantining procedure, which influence the policy in the patients to visit hospital since January 15, 2020. The vaccination period was defined as the time period since first AZ vaccination in Taiwan since Feb 15, 2021. In the beginning of vaccination in Taiwan, due to some sort of political issues, we did not gain the insufficient vaccination; the patients only receive two dosages of AZ, Moderna, and BNT without booster. This retrospective study was approved by the IRB (Ethical Committee of Taichung Veterans General Hospital No.CE22132A).

### Radiosurgical technique

After the patient had received a local anesthetic agent, the Leksell G head frame was affixed to the head, and the patient was monitored for blood pressure, oxygenation, and electrocardiography. Magnetic resonance imaging and MR angiography examination, including T1-weighted, T2 weighted, TOF, Spoiled gradient recall, and Gd enhanced sequence, were obtained to localize the lesions. In cases of intracranial AVM, results of MR imaging and cerebral angiography were transferred to the Leksell GammaPlan station (Elekta Instruments AB). Targets were delineated on the fused MR at various parameters or combined cerebral angiography images. The radiosurgery dose plans with single or multiple isocenters were created based on the contour of the target. All patients were treated with a Leksell Gamma Knife model D (Elekta AB) by a team consisting of a neurosurgeon, neuroradiologist, radiation oncologist, and medical physicist.

### Statistical analyses

Descriptive statistics were computed based on mean or median values. Factors contributing to neurological status, characteristics of lesions, time interval related to decision making and frequency of OPD visit and imaging follow-up and KPS were assessed with the Mann–Whitney test,* χ*^2^ test, and Fisher's Exact test. Logistic regression was used to assess risk factors related to the interval of decision making, frequency of OPD and MRI follow-up. Statistical significance was defined as a *p* < 0.05.

## Results

There were 207 cases before COVID-19 pandemic, and they served as the control. 185 cases were performed during the pandemic of COVID-19 without vaccination, and 148 cases performed during the pandemic with vaccination. Table 1 shows these patient parameters presented as mean ± standard deviation. The age of the patients was 55.9 ± 14.9 with ratio of male to female of 41.3%. There were 55.7% of the patients harboring neurological deficits and 36.3% with malignance. The gamma knife treatment parameter consisted of total treated volume of 16.2 ± 2.97 cc, peripheral dosage of 16.2 ± 5.2 Gy, and number of lesions with 1.6 ± 1.5. The parameters for analysis included KPS of 83.3 ± 8.1, ECOG of 0.8 ± 0.9, Charlson comorbidity index of 3.9 ± 3.2, time from the diagnosis to GK treatment of 44 ± 26.2 days, time from diagnosis to GK consultation of 22.5 ± 23.2 days, duration of GK treatment of 5.0 ± 1,4 h, OPD frequency of 10.4 ± 7.3/year, and MRI frequency of 2.0 ± 1.3/year.

**Table 1:** Characteristics of the patients

Parameters	Mean	SD
No COVID-19(n)	207(38.3%)	
COVID-19 and no vaccination(n)	185(34.3%)	
COVID-19 and vaccination(n)	148(27.4%)	
Age (years)	55.9	± 14.9
Sex-Male	223(41.3%)	
Neurological deficits (n, %)	301(55.7%)	
Malignance (n, %)	196(36.3%)	
Total TV (cc)	16.6	± 2.97
Peripheral dose (GY)	16.2	± 5.2
Number of lesions	1.6	± 1.5
KPS	83.8	± 8.1
ECOG	0.8	± 0.9
Charlson comorbidity index	3.9	± 3.2
Time from diagnosis to GK treatment (days)	44.0	± 26.2
Time from diagnosis to GK consultation (days)	22.5	± 23.2
Duration of GK treatment (hours)	5.0	± 1.4
OPD frequency (time/year)	10.4	± 7.3
MRI frequency (time/year)	2.0	± 1.3

Data were presented as mean ± standard deviation

TV, KPS, ECOG, OPD, MRI, GK: see abbreviation in text

Table 2 shows patient parameters according to the various treatment groups (Control, Pandemic, Vaccination). The data were presented as men ± standard deviation and measured by Kruskal–Wallis and* χ*^2^ test. We found a higher proportion of neurological deficits in the pandemic group (65.4%) compared with control (45.4%) or vaccination group (58.1%) (*p* < 0.001). The Charlson comorbidity index in three groups were 3.9 ± 3.3, 4.6 ± 3.2, and 3.1 ± 3.1, respectively, with significant intergroup difference (*p* < 0.001). Longer time intervals from diagnosis to GK consultation and GK treatment were found in the pandemic group (36.8 ± 25.5/54.5 ± 27.6 days) compared with control (17.1 ± 22.4/45.0 ± 28.0 days) and vaccination group (12.2 ± 7.1/29.6 ± 10.9 days) (all at *p* < 0.001). The accumulated time interval until GK treatment, frequency of OPD visiting, and the frequency of MRI examination also showed the same trend.

**Table 2:** Characteristics of patients sub-categorized by groups

	No COVID-19		COVID-19 and no vaccination		COVID-19 and vaccination		*p* value
Age (years)	54.8	± 15.0	56.4	± 14.9	56.6	± 15.0	0.454
Sex-Male (n,%)	87 (42%)		89 (48.1%)		47(31.8%)		0.010
Neurological deficits (n, %)	94 (45.4%)		121(65.4%)		86(58.1%)		< 0.001
Malignance (n, %)	83(40.1%)		64(34.6%)		49(33.1%)		0.337
Total TV (cc) (n = 538)	4.3	± 5.7	40.6	± 5.9	3.7	± 4.6	0.108
Peripheral dose (GY)	16.5	± 3.9	16.1	± 5.9	15.8	± 5.7	0.003
Number of lesions	1.9	± 1.9	1.5	± 1.3	1.4	± 1.1	0.011
KPS	83.1	± 8.5	83.9	± 7.3	84.7	± 8.4	0.208
ECOG	1.1	± 1.1	0.7	± 0.7	0.7	± 0.7	< 0.001
Charlson comorbidity index	3.9	± 3.3	4.6	± 3.2	3.1	± 3.1	< 0.001
Time from diagnosis to GK treatment (days)	45.0	± 28.0	54.5	± 27.6	29.6	± 10.9	< 0.001
Time from diagnosis to GK consultation (days)	17.1	± 22.4	36.8	± 25.5	12.2	± 7.1	< 0.001
Duration of GK treatment (hours)	4.8	± 1.4	5.1	± 1.4	5.2	± 1.4	0.007
OPD frequency (time/year)	13.7	± 7.6	6.9	± 6.0	10.0	± 6.3	< 0.001
MRI frequency (time/year)	2.6	± 1.1	1.0	± 1.0	2.3	± 1.0	< 0.001

Kruskal–Wallis test.* χ*^2^ test. Data were presented as mean ± standard deviation. TV, KPS, ECOG, OPD, MRI, GK: see abbreviation in text

Table 3 shows the influence of vaccination in the COVID-19 pandemic in GK decision behavior. The data were presented as men ± standard deviation and measured by Kruskal–Wallis test. The male predominance was shown in pandemic group related to vaccination group with the ratio of 48.1% to 31.7% (*p* < 0.01). There were significantly lower Charlson comorbidity indexes (3.1 ± 3.1/4.6 ± 3.2, *p* < 0.001), shorter time interval from diagnosis to GK consultation (12.1 ± 7.1/36.8 ± 25.2, *p* < 0.001), shorter time interval from diagnosis to GK treatment (29.6 ± 10.9/54.5 ± 27.6, *p* < 0.001) in the vaccination group. In contrast, increased frequencies in OPD visit (10.3 ± 6.3/6.9 ± 6.0, *p* < 0.001) and MRI examination (2.3 ± 1.0/1.0 ± 1/year, *p* < 0.001) were significantly higher in the vaccination group.

**Table 3:** Influence of vaccination on behaviors in GK decision during the pandemic period

	No (n = 185)		Yes (n = 148)		*p* value
	Mean	± SD	Mean	± SD	
Age (years)	56.4	± 14.9	56.6	± 15.0	0.958
Sex-Male (n, %)	89	(48.1%)	47	(31.7%)	0.004
Neurological deficits (n,%)	121	(65.4%)	86	(58.1%)	0.211
Malignance (n,%)	64	(34.6%)	49	(33.1%)	0.866
Total TV (cc) (n = 538)	40.6	± 507.9	3.7	± 4.6	0.318
Peripheral dose (GY)	16.1	± 5.9	15.8	± 5.7	0.086
Number of lesions	1.5	± 1.3	1.4	± 1.1	0.506
KPS	83.9	± 7.3	84.7	± 8.4	0.287
ECOG	0.7	± 0.7	0.7	± 0.7	0.377
Charlson comorbidity index	4.6	± 3.2	3.1	± 3.1	< 0.001
Time from diagnosis to GK treatment (days)	54.5	± 27.6	29.6	± 10.9	< 0.001
Time from diagnosis to GK consultation (days)	36.8	± 25.5	12.2	± 7.1	< 0.001
Duration of GK treatment (hours)	5.1	± 1.4	5.2	± 1.4	0.503
OPD frequency (time/year)	6.9	± 6.0	10.0	± 6.3	< 0.001
MRI frequency (time/year)	1.0	± 1.0	2.3	± 1.0	< 0.001

Mann–Whitney test. Data were presented as mean ± standard deviation

TV, KPS, ECOG, OPD, MRI, GK: see abbreviation in text

Table 4 shows the ratio of tumor volume change and increased number of lesions in MRI examination at time point of diagnosis and GK treatment. The data were presented as men ± standard deviation and measured by Kruskal–Wallis test. The increased ratio of tumor volume in three different groups was 5.25 ± 0.4%, 7.57 ± 0.52%, and 5.06 ± 0.27%, respectively (*p* < 0.05). The increased number of lesions among these different groups were 24.3 ± 3.52%, 36.5 ± 2.7%, and 26.2 ± 6.2%, respectively (*p* < 0.05). It indicated the hesitance in GK treatment augment the potential in increased tumor size and numbers.

**Table 4:** Increased volume and number of lesions of malignant tumor in MRI examination at the time points of diagnosis and GK treatment in three different groups

	No COVID-19	COVID-19 and no vaccination	COVID-19 and vaccination	*p* value
% of Increased tumor volume(n = number of lesions)	5.25 ± 0.4 (n = 201)	7.57 ± 0.52 (n = 104)	5.06 ± 0.27 (n = 89)	< 0.05
% of increased lesions (n = number of the patients)	24.3 ± 3.52 (n = 83)	36.5 ± 2.7 (n = 64)	26.2 ± 2.6 (n = 49)	< 0.05

Mann–Whitney test. Data were presented as mean ± standard deviation

The longer time interval from the diagnosis to GK consultation (21 days) was highly correlated with those in COVID-19 pandemic without vaccination as compared to the control or COVID-19 pandemic with vaccination (Additional file 1: Table 1S). The data were presented as men ± standard deviation and measured by Kruskal–Wallis and *χ*^2^ test. Those patients showed the longer interval from the diagnosis to GK consultation. with the characteristic of male predominance (48.5%/37.9%, *p* < 0.05), no malignance (18.1%/44.7%, *p* < 0.001), and fewer numbers of lesion (1.4 ± 1.3/1.7 ± 1.6, *p* < 0.01).

The time interval (> 45 days) from diagnosis to GK treatment was also highly correlated with those in COVID-19 pandemic relative to the other two groups (Additional file 1: Table 2S). The data were presented as men ± standard deviation and measured by Kruskal–Wallis and *χ*^2^ test. Those patients with malignance (45.2%/18.1%, *p* < 0.001), higher peripheral dosage (16.7 ± 5.6/15.1 ± 3.8 Gy, *p* < 0.001), more number of lesions (1.7 ± 1.6/1.4 ± 1.2, *p* < 0.01), and high Charlson comorbidity index (4.4 ± 3.4/3.0 ± 2.7, *p* < 0.001) showed the shorter interval from the diagnosis to GK treatment.

The longer gamma knife treatment time (> 5.5 h) was also found in the COVID period group and also in patients with higher Charlson comorbidity index (Additional file 1: Table 3S). The data were presented as men ± standard deviation and measured by Kruskal–Wallis and *χ*^2^ test. Those patients with neurological deficits (63.7%/53.1%, *p* < 0.05), malignance (40.5%/23.7%, *p* < 0.001), larger total treated volume (56.3 ± 5.94/3.4 ± 4.9 cc, *p* < 0.001), and higher Charlson comorbidity index (4.2 ± 3.3/3.1 ± 3.0, *p* < 0.001) showed the longer GK treatment time.

OPD visiting (> 14/years) in the COVID-19 status were fewer when compared with the control or vaccination group (Additional file 1: Table 4S). The data were presented as men ± standard deviation and measured by Kruskal–Wallis and *χ*^2^ test. The increased OPD visits were also present in the patients with older age (60 ± 11.8/54.1 ± 15.8, *p* < 0.001), no neurological deficits(41.6%/61.8%, *p* < 0.001), malignance (81.4%/17.2%, *p* < 0.001), larger targeted volume (46.2 ± 5.44/4 ± 4.9, *p* < 0.001), larger peripheral radiation dose(19.1 ± 5.5/14.9 ± 4.5, *p* < 0.001), more number of lesions (2.5 ± 2.3/1.2 ± 0.7, p < 0.001), greater KPS (80.7 ± 8.6/85.1 ± 7.5, *p* < 0.001), greater ECOG (1.2 ± 1.2/0.7 ± 0.7, *p* < 0.001), and high Charlson comorbidity indices (7.4 ± 2.4/2.5 ± 2.3, *p* < 0.002). The analysis for fewer MRI examinations is shown in Additional file 1: Table 5S. The data were presented as men ± standard deviation and measured by Kruskal–Wallis and *χ*^2^ test. Risk factors analyses also revealed the same trend in frequency of OPD visit such as older age (60.3 ± 12.3/54.3 ± 15.5, *p* < 0.001), no neurological deficits (34.3%/63.5%, p < 0.001), malignancy (87.4%/18.0%, *p* < 0.001), total target volume (21.6 ± 3.48/3.2 ± 5.2 cc, *p* < 0.01), peripheral radiation dose (18.9 ± 2.4/15.2 ± 5.5 Gy, *p* < 0.001), increased number of lesions (2.5 ± 2.3/1.3 ± 0.9, *p* < 0.001), lower KPS (79.9 ± 8.3/85.2 ± 7.5, *p* < 0.001), higher ECOG (1.3 ± 1.2/0.7 ± 0.7, *p* < 0.001), and higher Charlson comorbidity index (7.3 ± 2.1/2.7 ± 2.7, *p* < 0.001).

**Fig. 1:**
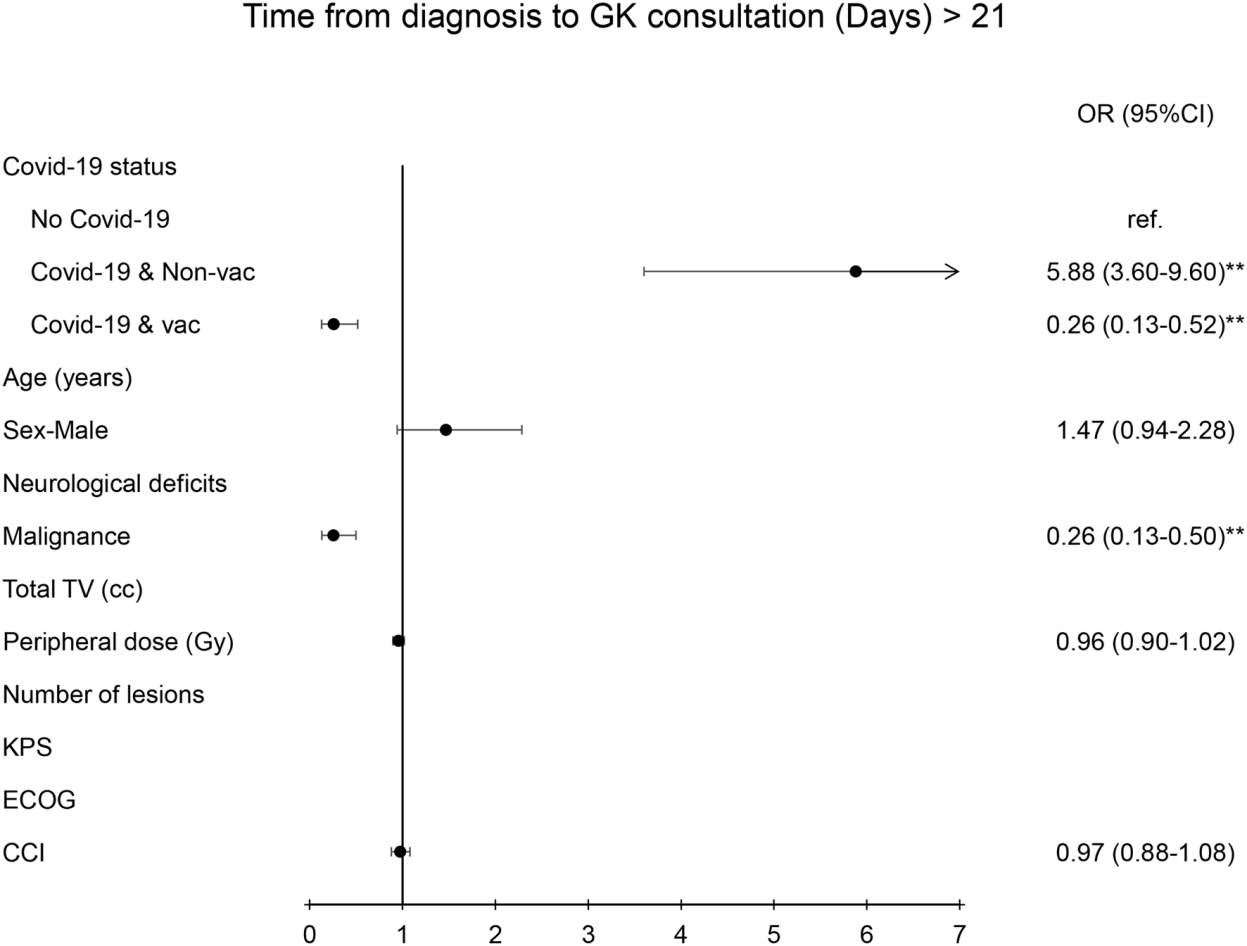
Diagram illustration of odds ratio of time interval from the diagnosis to GK consultation (> 21 days). Left side panel presented the parameters of risk factors. Right side panel presented the odds ratio with 95% confidence interval. ** indicated *p* value < 0.01. TV, KPS, ECOG, CCI, OR: see abbreviation in text

**Fig. 2:**
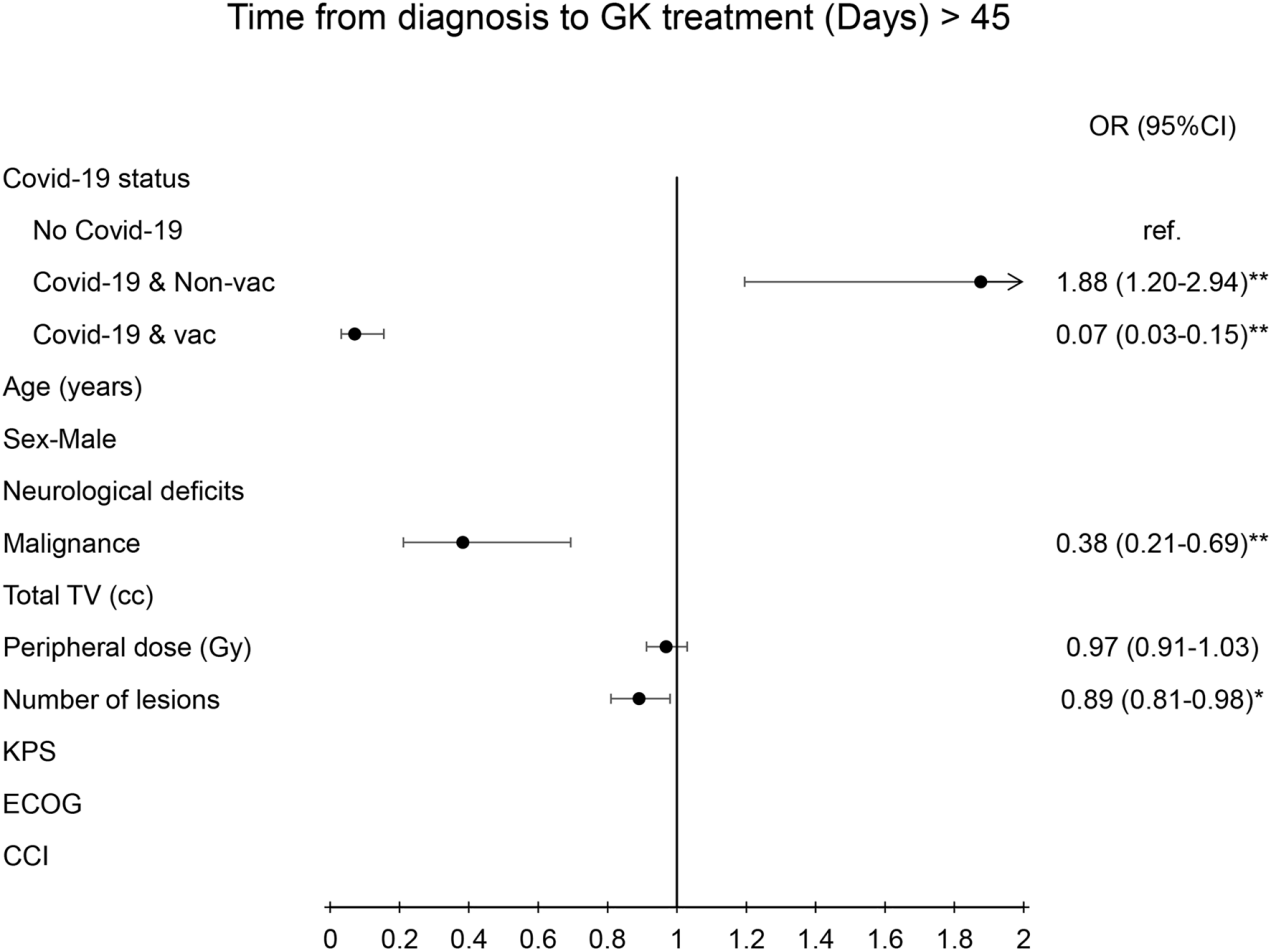
Diagram illustration of odds ratio of time interval from the diagnosis to GK treatment (> 45 days). Left side panel presented the various risk factors. Right side panel presented the odds ratio with 95% confidence interval. * indicated *p* value < 0.05; ** indicated *p* value < 0.01. TV, KPS, ECOG, CCI, OR: see abbreviation in text

In logistic regression with multi-variate analysis, the determining factors that contributed to longer time intervals from diagnosis to consultation were COVID status (OR = 5.88(3.6–9.6), *p* < 0.01) and the status of the malignant tumor (OR = 0.26(0.13–0.15), *p* < 0.01) (Fig. 1). The critical factors contributed to longer time intervals from diagnosis to GK treatment were COVID status (OR-1.88(1.2–2.94), *p* < 0.01), status of the malignant tumor (OR = 0.38(0.21–0.69), *p* < 0.01), and number of lesions (OR = 0.89(0.81–0.98, *p* < 0.01) (Fig. 2).

**Fig. 3:**
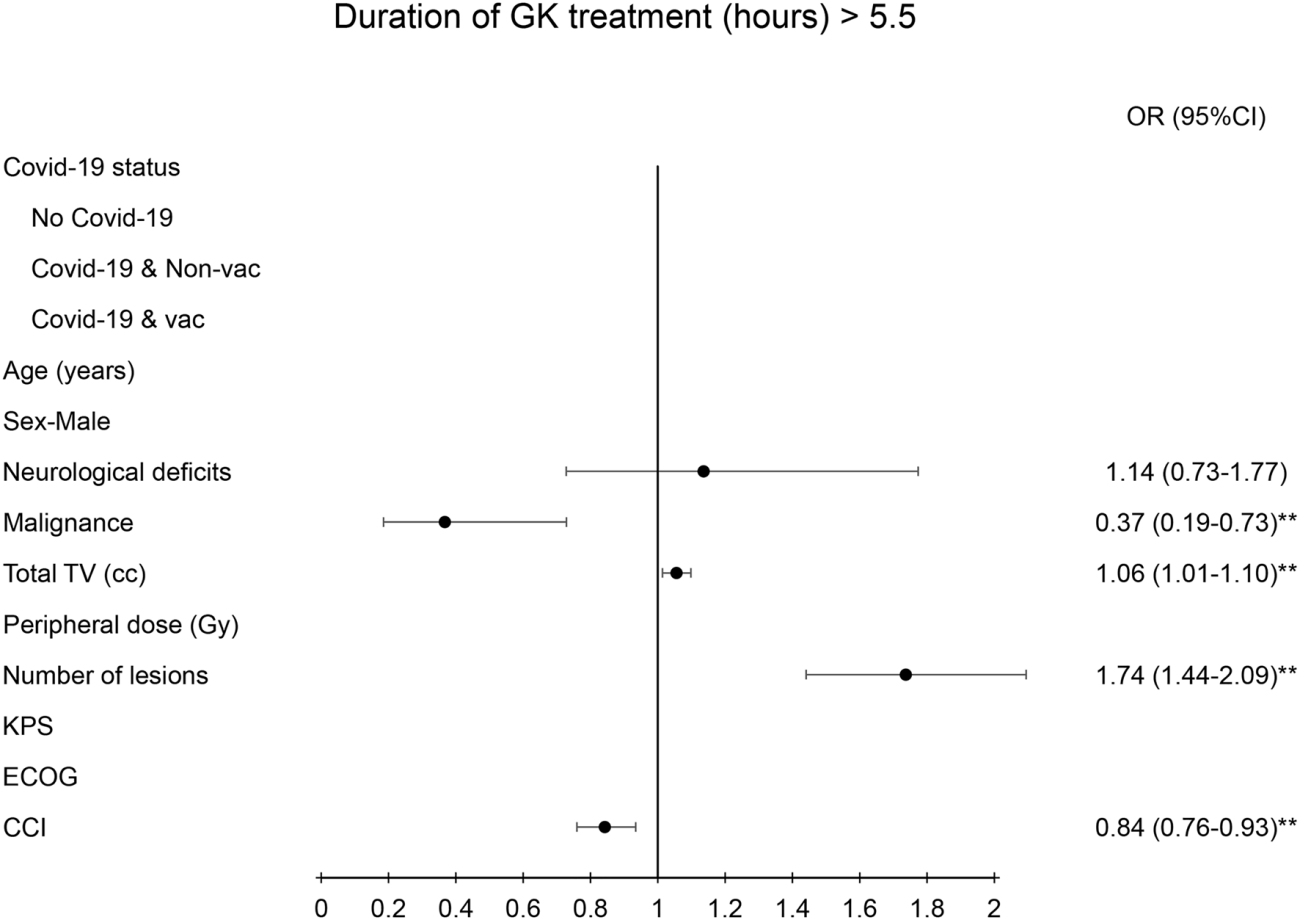
Diagram illustration of odds ratio of duration of GK treatment (> 4.5 h). The risk factors were presented in the Left side panel. Right side panel presented the odds ratio with 95% confidence interval. ** indicated *p* value < 0.01. TV, KPS, ECOG, CCI, OR: see abbreviation in text

**Fig. 4:**
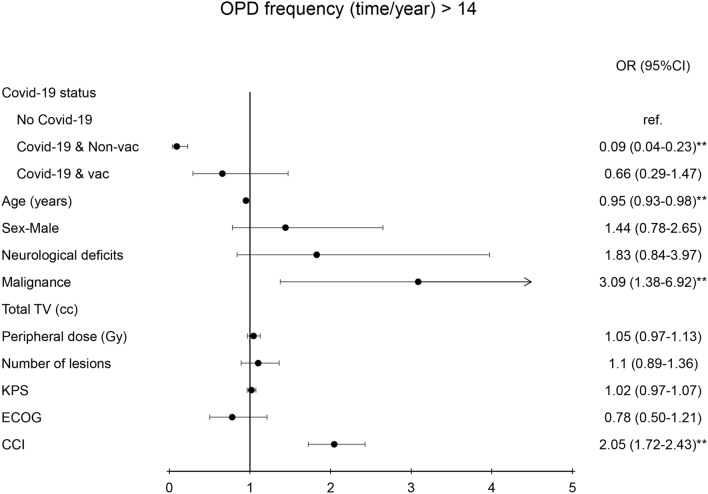
Diagram illustration of odds ratio of the frequency of outpatient department (OPD) visiting (> 14/year). Left side panel presented the parameters of the risk factors. Right side panel presented the odds ratio with 95% confidence interval. ** indicated *p* value < 0.01. TV, KPS, ECOG, CCI, OR: see abbreviation in text

**Fig. 5:**
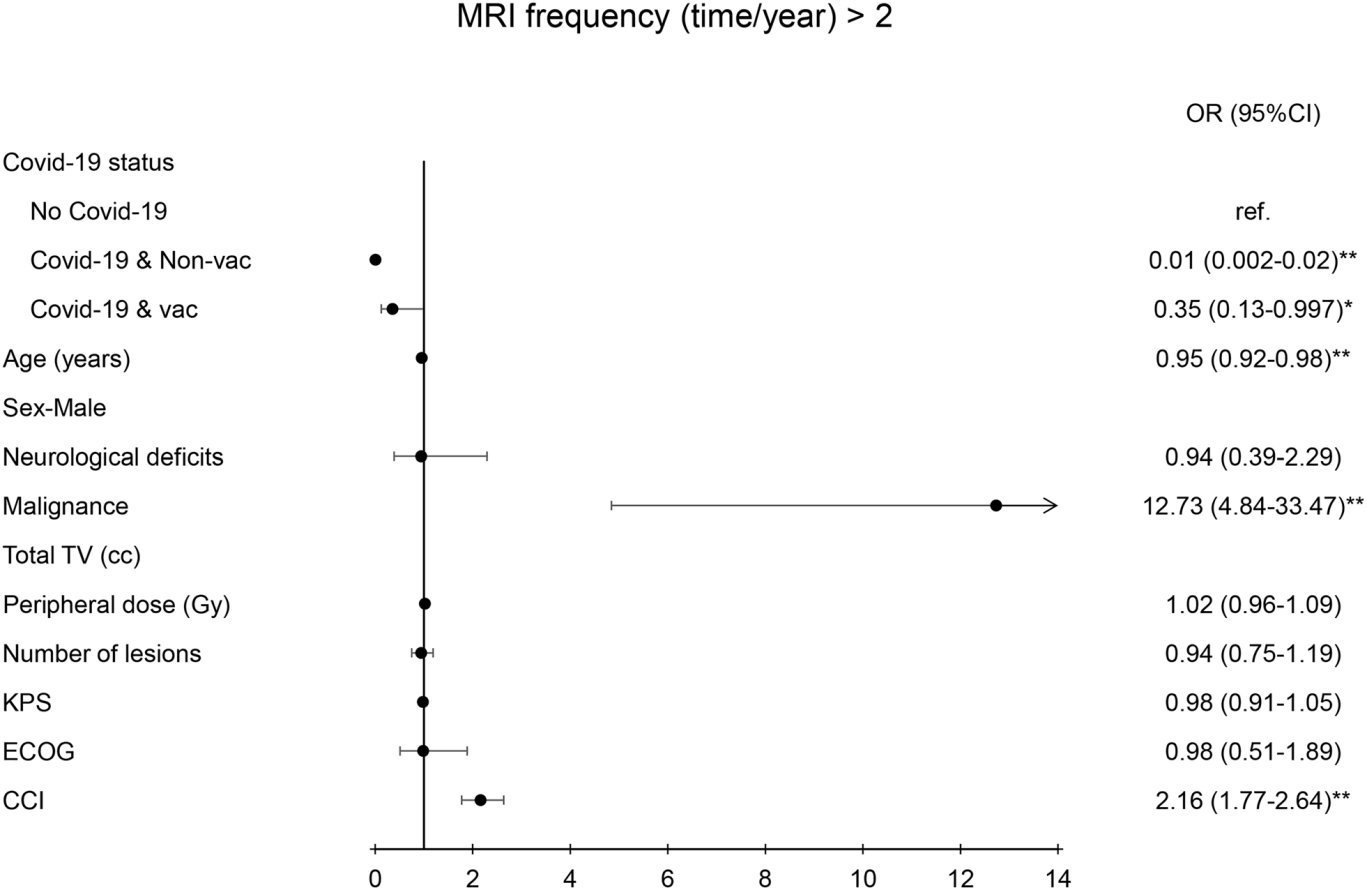
Diagram illustration of odds ratio of the frequency of MRI examination (> 2/year). Left side panel showed the risk factors. Right side panel presented the odds ratio with 95% confidence interval. ** indicated *p* value < 0.01. TV, KPS, ECOG, CCI, OR: see abbreviation in text

The treatment time during the GK procedure was highly correlated with the status of malignancy (OR = 0.37(0.19–0.73), *p* < 0.01), total treated volume (OR = 1.06 (1.01–1.1), *p* < 0.01), number of lesions (OR = 1.74 (1.44–2.09), *p* < 0.01), and Charlson comorbidity index (OR = 0.84 (0.76–0.93), *p* < 0.01) (Fig. 3). Fewer OPD visit were highly correlated with the status of COVID-19 (OR = 0.09(0.04–0.23), *p* < 0.01), while old age (OR = 0.95(0.93–0.98), *p* < 0.01), malignance (OR = 3.09(1.38–6.92), *p* < 0.01), and Charlson comorbidity index (OR = 2.05 (1.72–2.43), *p* < 0.01) predisposed patients to a higher frequency in OPD visit (Fig. 4). The lower frequency of MRI examination was also correlated with the status of COVD-19 (OR = 0.01(0.002 = 0.02), *p* < 0.01), while old age (OR = 0.95 (0.92–0.98), *p* < 0.01), malignancy (OR = 12.73(4.84–33.73), *p* < 0.01), and high Charlson comorbidity index (OR = 2.16(1.77 = 2.64), *p* < 0.01) had a tendency of having more MRI examination (Fig. 5).

## Discussion

Gamma knife treatment is an effective tool for many intracranial lesions, but typically it affords a delayed therapeutic response. Hence, the decision making for the patients to receive gamma knife treatment could be influenced by catastrophic events such as COVID-19 pandemics either with or without vaccination protection for patients. In this study, we confirmed the time interval from diagnosis to neurosurgical consultation or GK treatment occurred in a delayed fashion, and the delay in decision making was shortened by the patient vaccination when available. Those patients with neurological deficit or high Charlson comorbidity index received gamma knife treatment without hesitance. Thus, patients’ predilection for gamma knife treatment should be adjusted during the COVID-19 pandemic, but the hesitance could be tailored by COVID vaccination.

Charlson comorbidity index is well-known for predicting the risk of mortality based on medical records [17]. In general, high comorbidity index is associated with short survival and high mortality in the patients with malignant tumor [18, 19]. As expected, during COVID-19 pandemic, a high proportion of the patients with high Charlson comorbidity indices underwent gamma knife radiosurgery, whereas during non-COVID, or with vaccination, scores in CCI index returned to normal. In addition, those patients with high Charlson comorbidity were defined as high-risk groups to get COVID-19 infection and suggested to stay at home except at the condition of illness required for the medical service. Therefore, those with high Charlson comorbidity patients hesitate to visit the clinics and also afraid to receive gamma knife treatment due to the vulnerability to get COVID-19 infection. Therefore, the patients with the higher Charlson comorbidity were mainly existed in the control group. The high Charlson comorbidity is reversely correlated with the ECOG and KPS level. However, according to our policy of gamma knife treatment, a KPS > 70 or ECOG < 1 was a prerequisite for approval of radiosurgical treatment. Thus, the required condition may have dampened paralleling trends in patients undergoing gamma knife treatment.

We found significantly more incidence of metastasis by 30% in patients with MRI imaging before and at the time point of gamma knife radiosurgery. Such increase reached to 85% in case of hepatoma [20]. In our study, the significantly increased tumor volume was observed in the COVID-19 pandemic compared to the control group, but significantly reduced after the vaccination. In addition, the significantly increased in lesion number was also found in pandemic group relative to control group. The increased trend was also attenuated by vaccination. Due to volumetric errors largely influenced by the slice number and tumor morphology, the data errors may reach > 10% when the number of MRI slices less than 10 [21, 22]. In this study, we did not assure that the hesitance in GK treatment really promoted tumor growth due to slight increase in tumor volume counteracted by the measurement errors. However, the increased number of lesions in metastatic tumor during the pandemic period really happened. Thus, the status of COVID-19 certainly affected the decision making in the patients requiring the gamma knife treatment, which may aggravate the disease progression. For those patients harboring the malignance, the hesitance in accepting such treatment should be attenuated and also modulated by healthcare policy.

The frequency of OPD visit and the number of MRI imaging reflected patient’s status related to regular medication, laboratory assessment, and neurological condition. In this study, significantly fewer OPD visit and fewer MRI imaging were found in the group of COVID-19 status when compared with the pre-COVID status or with the periods after vaccination availability. Such change was not altered in those patients with malignant tumors. Findings indicated that decision making in OPD visit or MRI examination was influenced by the emergence of COVID-19 and attenuated by vaccination administration. The healthcare policy may also change the decision making in the patients related to medical issue in gamma knife treatment. After the COVID-19 pandemic, the institute for approval for gamma knife treatment was shifted from section institute of Taiwan NHI to the individual hospital management department. The penalty for medical expense above the estimated individual hospital budge was also exempted, which may reduce the waiting time for the patient to receive MRI examination.

The presence of neurological deficits was a warning sign for patients to make visits to the neurological department for determining the pathology of the disease. As expected, those patients with the neurological deficits after the gamma knife approval promptly accepted treatment without hesitance. In this study, in the case of having neurological deficits, the decision making for gamma knife treatment was not influenced by the COVID pandemic.

The decision making for the patients undergoing gamma knife treatment was most likely influenced by the COVID-19 pandemic and also affected by the emergence of vaccinations. There were several limitations in this study. Decision making is a complex process not merely based on the time interval from the diagnosis to consultation or treatment but also the frequencies of OPD and follow-up brain MRI assessment. The change of healthcare policy during the pandemic period also partially contributed to the decision making by the patients. To better clarify the decision making in receiving gamma knife treatment, double blind and randomized studies need to be conducted.

## Conclusions

The decision making in the patients receiving gamma knife treatment appeared appreciably influenced by the pandemic COVID-19. Those patients harboring an intracranial malignancy or the neurological deficits were not influenced in a similar way.

## Supplementary Information

**Additional file 1: Table 1S.** Risk factors analysis for the patient with longer time interval from diagnosis to GK consultation (>21 days, anterior tripartition). **Table 2S.** Risk factors analysis for the patient with longer time interval from diagnosis to GK treatment (>45 days, anterior tripartition). **Table 3S.** Risk factors analysis for the patient with longer time interval during GK treatment (>5.5 h, anterior tripartition). **Table 4S.** Risk factors analysis for the patient with increased OPD frequency (>14 times/year, anterior tripartition). **Table 5S.** Risk factors analysis for the patient with increased MRI frequency (>2 times/year, anterior tripartition).

## Data Availability

The authors confirm that the data supporting the findings of this study are available within the article and its supplementary materials.

## Abbreviations

GK: Gamma knife radiosurgery
KPS: Karnofsky Performance Status
MRI: Magnetic Resonance Imaging
TOF: Time-of-flight
OPD: Outpatient department
ECOG: Eastern Cooperative Oncology Group Performance Status
TV: Treated volume
Gy: Gray
CCI: Charlson comorbidity index
OR: Odds ratio
AZ: AstraZeneca vaccine
BNT: BioNTech
