# Supplementary material for: Influence of COVID-19 pandemic on the decision making of patients in undergoing gamma knife radiosurgery
Source: Eur J Med Res. 2022 Oct 29;27:223. doi: 10.1186/s40001-022-00859-w (PMC9617744; doi:10.1186/s40001-022-00859-w)
Supplement: Supplementary file 1 — Additional file 1: Table 1S. Risk factors analysis for the patient with longer time interval from diagnosis to GK consultation (>21 days, anterior tripartition). Table 2S. Risk factors analysis for the patient with longer time interval from diagnosis to GK treatment (>45 days, anterior tripartition). Table 3S. Risk factors analysis for the patient with longer time interval during GK treatment (>5.5 h, anterior tripartition). Table 4S. Risk factors analysis for the patient with increased OPD frequency (>14 times/year, anterior tripartition). Table 5S. Risk factors analysis for the patient with increased MRI frequency (>2 times/year, anterior tripartition). [file 40001_2022_859_MOESM1_ESM.pdf]

Supplementary data

Table 1S: Risk factors analysis for the patient with longer time interval from diagnosis to GK consultation (>21days, anterior tripartition)

	No (n=369)		Yes (n=171)		*p* value
	Mean	±SD	Mean	±SD	
Covid-19 status & vaccination (n, %)					<0.001
No Covid-19	160	(43.4%)	47	(27.5%)	
Covid-19 & No vaccination	73	(19.8%)	112	(65.5%)	
Covid-19 & vaccination	136	(36.9%)	12	(7.0%)	
Age (years)	56.1	±14.5	55.3	±15.9	0.530
Sex-Male (n, %)	140	(37.9%)	83	(48.5%)	0.026
Neurological deficits (n, %)	198	(53.7%)	103	(60.2%)	0.181
Malignance (n, %)	165	(44.7%)	31	(18.1%)	<0.001
Total TV (cc)	22.8	±360.6	3.5	±5.0	0.545
Peripheral dose (GY)	16.7	±5.8	15.0	±3.3	0.001
Number of lesions	1.7	±1.6	1.4	±1.3	0.003
KPS	83.6	±8.5	84.4	±7.3	0.408
ECOG	0.9	±1.0	0.8	±0.7	0.312
Charlson comorbidity index	4.2	±3.4	3.4	±2.9	0.066

Mann-Whitney test. Chi-Square test. Data was presented as mean ± standard deviation. TV, KPS, ECOG: see abbreviation in text.

Table 2S: Risk factors analysis for the patient with longer time interval from diagnosis to GK treatment (>45 days, anterior tripartition)

	No (n=363)		Yes (n=177)		*p* value
	Mean	±SD	Mean	±SD	
Covid-19 status & vaccination (n, %)					<0.001
No Covid-19	131 (36.1%)		76 (42.9%)		
Covid-19 & No vaccination	92 (25.3%)		93 (52.5%)		
Covid-19 & vaccination	140 (38.6%)		8 (4.5%)		
Age (years)	56.6	±14.6	54.3	±15.5	0.070
Sex-Male (n, %)	147 (40.5%)		76 (42.9%)		0.654
Neurological deficits (n, %)	198 (54.5%)		103 (58.2%)		0.479
Malignance (n, %)	164 (45.2%)		32 (18.1%)		<0.001
Total TV (cc)	22.9	±363.6	3.9	±4.8	0.381
Peripheral dose (GY)	16.7	±5.6	15.1	±3.8	<0.001
Number of lesions	1.7	±1.6	1.4	±1.2	<0.001
KPS	83.4	±8.5	84.6	±7.2	0.220
ECOG	0.9	±0.9	0.8	±0.8	0.343
Charlson comorbidity index	4.4	±3.4	3.0	±2.7	<0.001

Mann-Whitney test. Chi-Square test. Data was presented as mean ± standard deviation. TV, KPS, ECOG: see abbreviation in text.

Table 3S: Risk factors analysis for the patient with longer time interval during GK treatment (>5.5 hours, anterior tripartition)

	No (n=405)		Yes (n=135)		*p* value
	Mean	±SD	Mean	±SD	
Covid-19 status & vaccination (n, %)					0.266
No Covid-19	163 (40.2%)		44 (32.6%)		
Covid-19 & No vaccination	133 (32.8%)		52 (38.5%)		
Covid-19 & vaccination	109 (26.9%)		39 (28.9%)		
Age (years)	56.4	±15.0	54.3	±14.9	0.103
Sex-Male (n, %)	173 (42.7%)		50 (37.0%)		0.289
Neurological deficits (n, %)	215 (53.1%)		86 (63.7%)		0.040
Malignance (n,%)	164 (40.5%)		32 (23.7%)		0.001
Total TV (cc)	3.4	±4.9	56.3	±5.94	<0.001
Peripheral dose (GY)	16.2	±4.6	16.0	±6.6	0.222
Number of lesions	1.4	±1.0	2.1	±2.4	0.183
KPS	83.8	±8.3	84.1	±7.4	0.692
ECOG	0.8	±0.8	0.9	±1.2	0.948
Charlson comorbidity index	4.2	±3.3	3.1	±3.0	<0.001

Mann-Whitney test. Chi-Square test. Data was presented as mean ± standard deviation. TV, KPS, ECOG: see abbreviation in text.

Table 4S: Risk factors analysis for the patient with increased OPD frequency (>14 times/year, anterior tripartition)

	No (n=377)		Yes (n=161)		*p* value
	Mean	±SD	Mean	±SD	
Covid-19 status & vaccination (n, %)					0.002
No Covid-19	126 (33.4%)		80 (49.7%)		
Covid-19 & No vaccination	141 (37.4%)		43 (26.7%)		
Covid-19 & vaccination	110 (29.2%)		38 (23.6%)		
Age (years)	54.1	±15.8	60.1	±11.8	<0.001
Sex-Male (n, %)	145 (38.5%)		77 (47.8%)		0.054
Neurological deficits (n, %)	233 (61.8%)		67 (41.6%)		<0.001
Malignance (n, %)	65 (17.2%)		131 (81.4%)		<0.001
Total TV (cc)	4.0	±4.9	46.2	±5.44	0.001
Peripheral dose (GY)	14.9	±4.5	19.1	±5.5	<0.001
Number of lesions	1.2	±0.7	2.5	±2.3	<0.001
KPS	85.1	±7.5	80.7	±8.6	<0.001
ECOG	0.7	±0.7	1.2	±1.2	<0.001
Charlson comorbidity index	2.5	±2.3	7.4	±2.4	<0.001

Mann-Whitney test. Chi-Square test. Data was presented as mean ± standard deviation. TV, KPS, ECOG: see abbreviation in text.

Table 5S: Risk factors analysis for the patient with increased MRI frequency (>2 times/year, anterior tripartition)

	No (n=395)		Yes (n=143)		*p* value
	Mean	±SD	Mean	±SD	
Covid-19 status & vaccination (n, %)					<0.001
No Covid-19	120 (30.4%)		86 (60.1%)		
Covid-19 & No vaccination	165 (41.8%)		19 (13.3%)		
Covid-19 & vaccination	110 (27.8%)		38 (26.6%)		
Age (years)	54.3	±15.5	60.3	±12.3	<0.001
Sex-Male (n, %)	159 (40.3%)		63 (44.1%)		0.489
Neurological deficits (n,%)	251 (63.5%)		49 (34.3%)		<0.001
Malignance (n,%)	71 (18.0%)		125 (87.4%)		<0.001
Total TV (cc)	21.6	±3.48	3.2	±5.2	0.002
Peripheral dose (GY)	15.2	±5.5	18.9	±2.4	<0.001
Number of lesions	1.3	±0.9	2.5	±2.3	<0.001
KPS	85.2	±7.5	79.9	±8.3	<0.001
ECOG	0.7	±0.7	1.3	±1.2	<0.001
Charlson comorbidity index	2.7	±2.7	7.3	±2.1	<0.001

Mann-Whitney test. Chi-Square test. Data was presented as mean ± standard deviation.TV, KPS, ECOG: see abbreviation in text.
